# Supplementary material for: The origins and genetic interactions of KRAS mutations are allele- and tissue-specific
Source: Nat Commun. 2021 Mar 22;12:1808. doi: 10.1038/s41467-021-22125-z (PMC7985210; doi:10.1038/s41467-021-22125-z)
Supplement: Supplementary file 1 — Supplementary Information [file 41467_2021_22125_MOESM1_ESM.pdf]

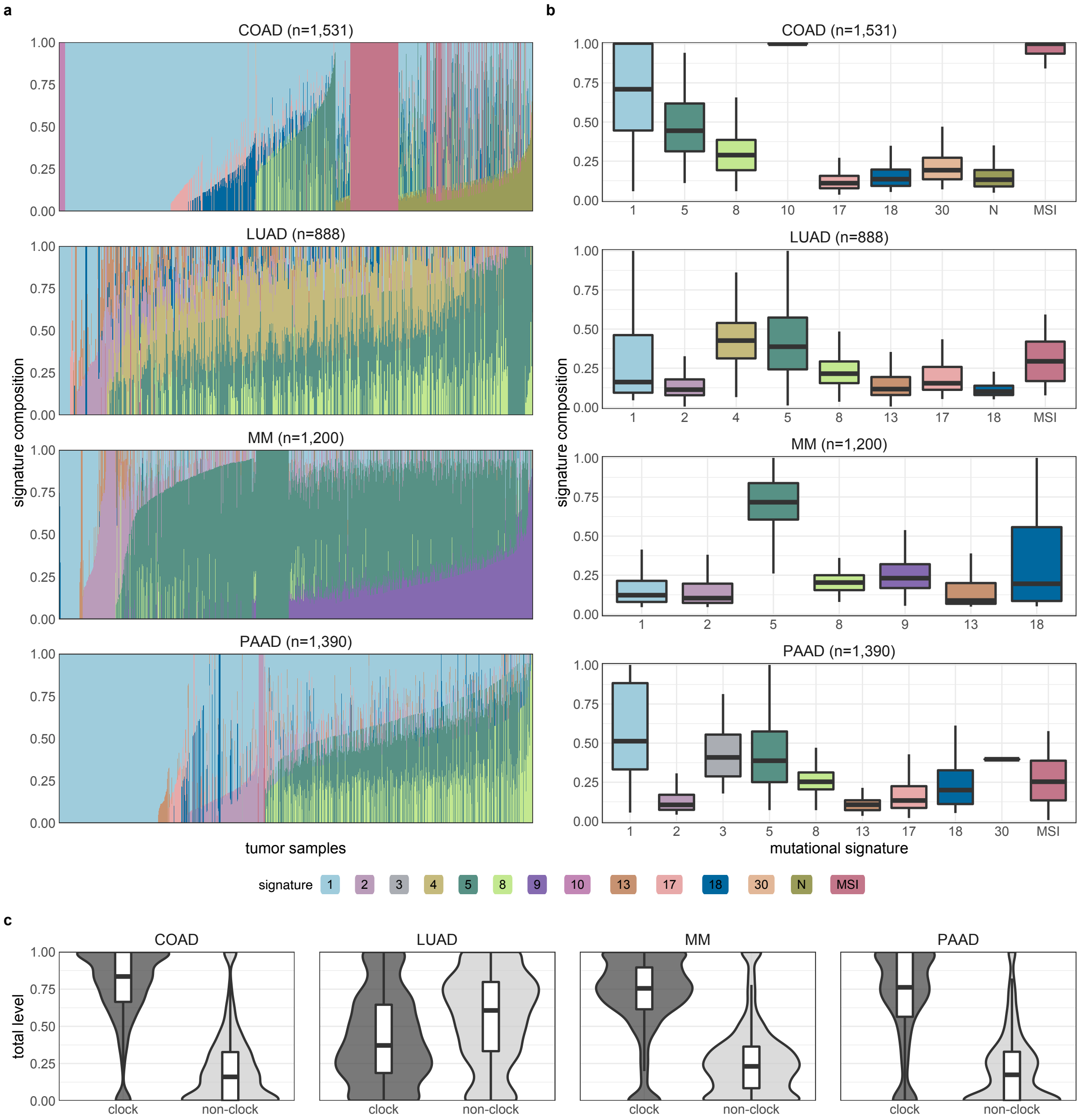

Supplementary Figure 1. Mutational signatures in tumor samples. a. The composition of the mutational signatures in each tumor sample, with each column representing a tumor sample. **b**. The distributions of the fraction of total mutational signature composition per tumor sample for each signature detected in the cancer type. **c.** The average levels of clock (signatures 1 and 5) and non-clock (all other signatures) in the tumor samples. For the box plots, the box demarcations represent the 25th, 50th, and 75th percentiles, and the whiskers extend from the box to the largest and smallest data points at most 1.5 times the inter-quartile range away from the median.

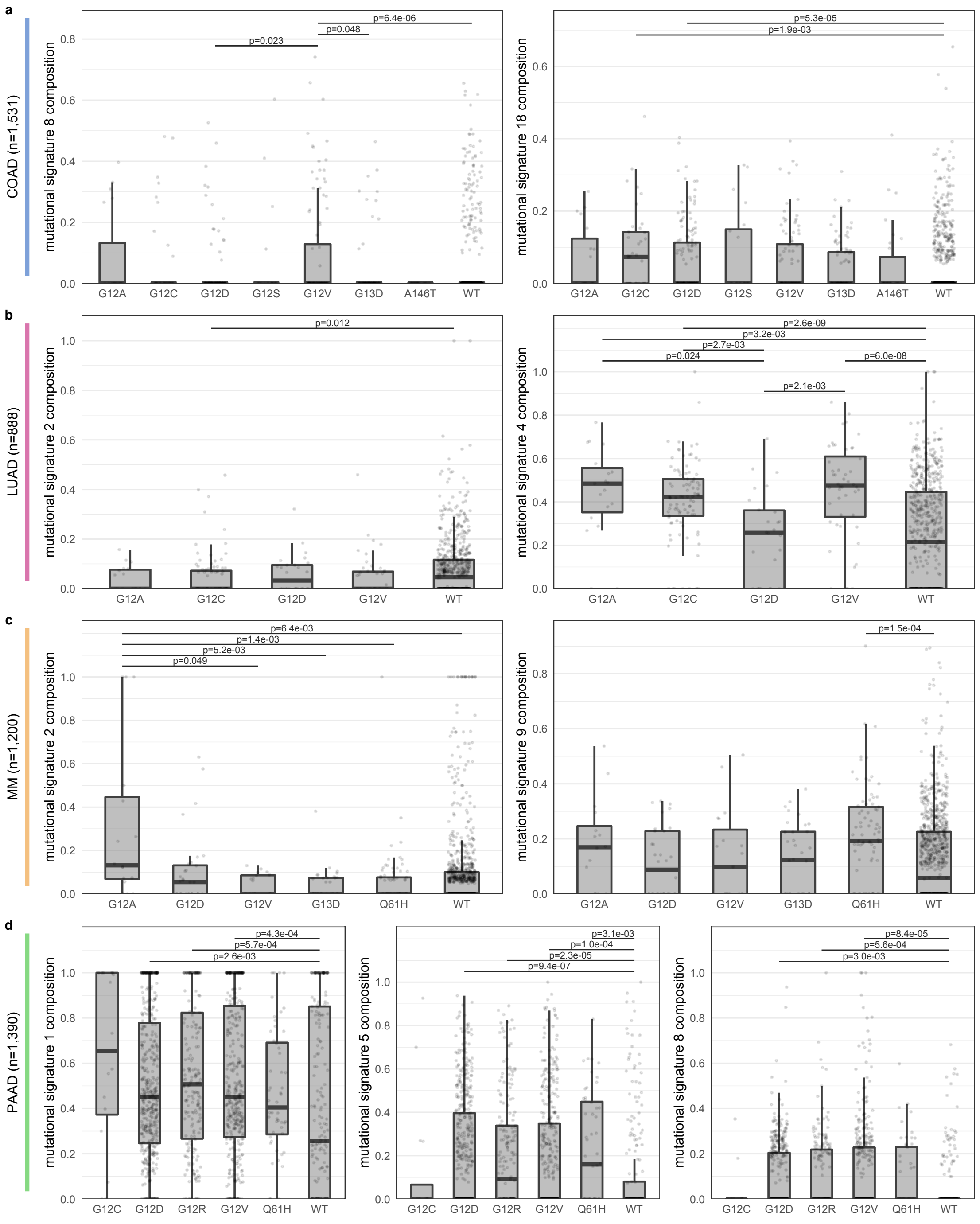

Supplementary Figure 2. Mutational signature compositions by *KRAS* allele. The composition of select mutational signatures in (**a**) COAD, (**b**) LUAD, (**c**) MM, or (**d**) PAAD tumor samples separated by their *KRAS* allele. Each point represents an individual tumor sample. Bars above each box-plot indicate statistically significant differences between *KRAS* alleles (Wilcoxon rank-sum test; p-values were adjusted using the Bonferroni method). Any mutational signature with at least one statistically significant difference is presented, except for mutational signatures with consistently very low levels in the cancer. For the box plots, the box demarcations represent the 25th, 50th, and 75th percentiles, and the whiskers extend from the box to the largest and smallest data points at most 1.5 times the inter-quartile range away from the median.

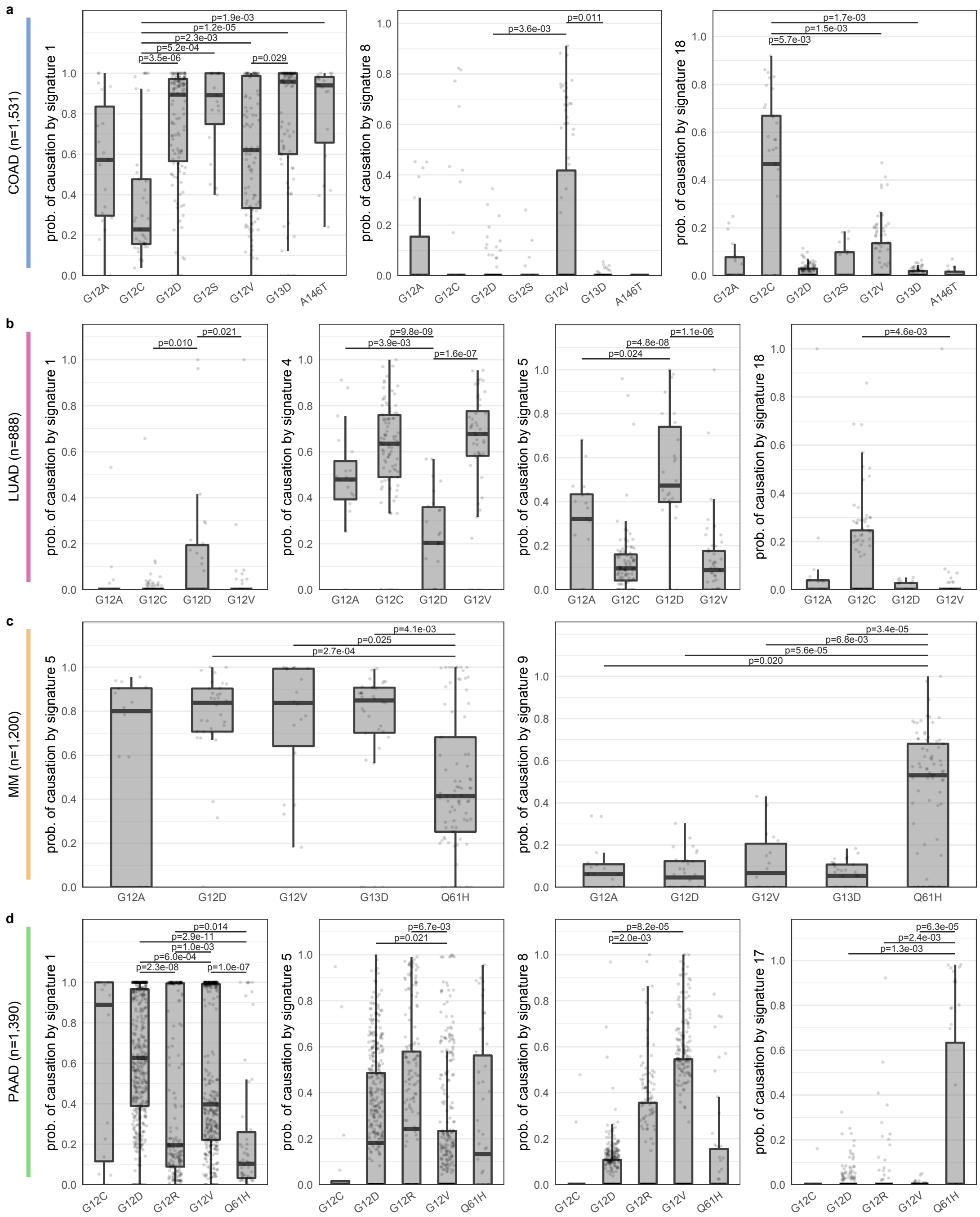

Supplementary Figure 3. Distributions of the probabilities of mutational signatures to have caused *KRAS* mutations. The probability of select mutational signatures to have caused the indicated *KRAS* mutations in (**a**) COAD, (**b**) LUAD, (**c**) MM, or (**d**) PAAD tumor samples. Each point represents an individual tumor sample. Bars above each box-plot indicate statistically significant differences between *KRAS* alleles (Wilcoxon rank-sum test; p-values were adjusted using the Bonferroni correction method). Any mutational signature with at least one statistically significant difference is presented, except for mutational signatures with consistently very low probabilities of causing the *KRAS* mutations in the cancer. For the box plots, the box demarcations represent the 25th, 50th, and 75th percentiles, and the whiskers extend from the box to the largest and smallest data points at most 1.5 times the inter-quartile range away from the median.

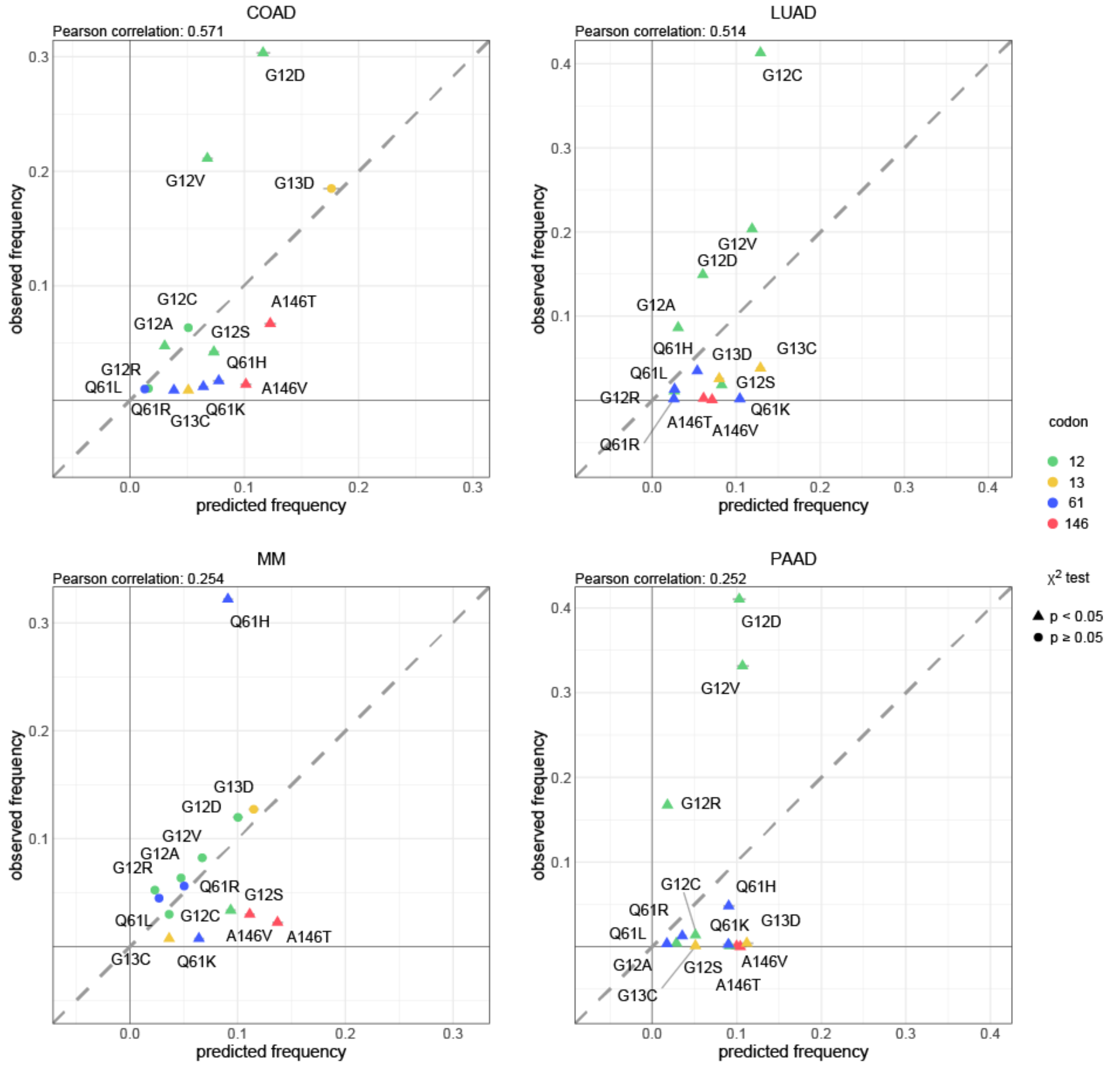

Supplementary Figure 4. The predicted frequencies of all oncogenic *KRAS* alleles in each cancer. The predicted vs. observed frequency of *KRAS* alleles in each cancer for all *KRAS* alleles demonstrated to drive any of the four cancers. The *KRAS* alleles included in the calculation were found mutated frequently in at least one of the four cancer types. Triangles indicate rejection of the null hypothesis that the observed and predicted frequencies are the same (χ-squared test, FDR-adjusted p-value < 0.05). Circles indicate the failure to reject the null hypothesis (χ-squared test, FDR-adjusted p-value ≥ 0.05). Error bars indicate bootstrapped 95% confidence intervals of the predicted values.

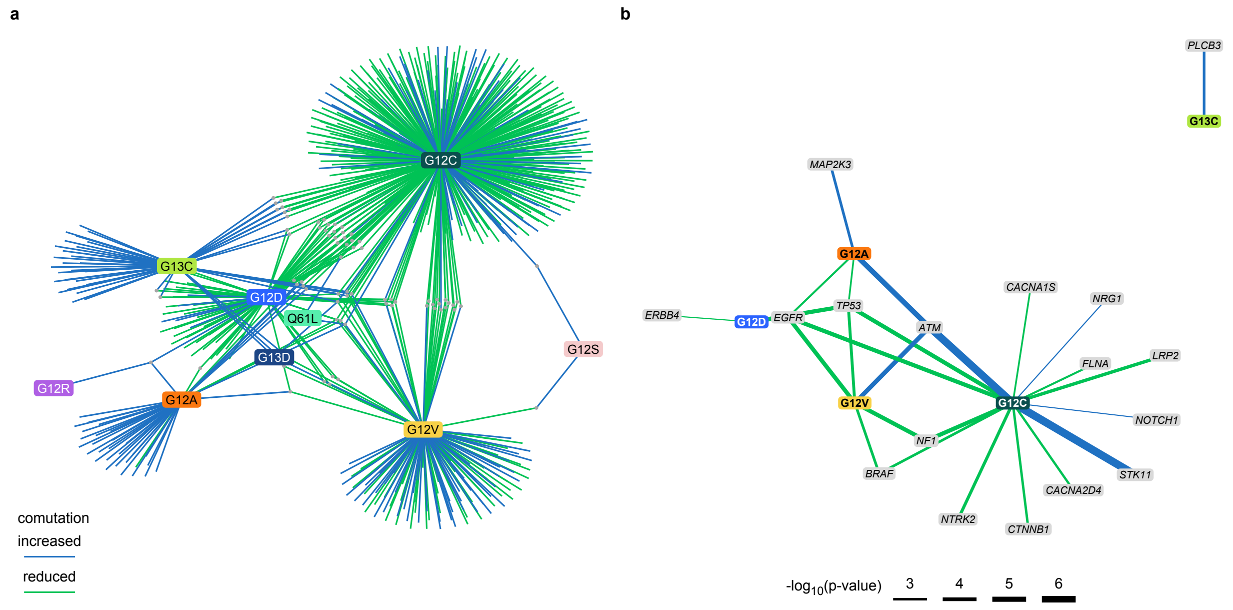

Supplementary Figure 5. The comutation networks of *KRAS* alleles in LUAD. a. The significant comutation network of the *KRAS* alleles in LUAD where each edge represents a comutation interaction between an allele and another gene (p-value < 0.01). The color of the edge indicates whether the interaction was an increase (blue) or decrease (green) in the frequency of comutation. Genes with multiple interactions are represented by a grey dot to disambiguate them from where edges intersect. **b.** A subset of the network shown in a of genes in one of the canonical up- or downstream signaling pathways of K-RAS. The width of the edge indicates the strength of the association. n = 5,051 biologically independent LUAD tumor samples for the increased comutation analysis, and n = 891 biologically independent LUAD tumor samples for the reduced comutation analysis.

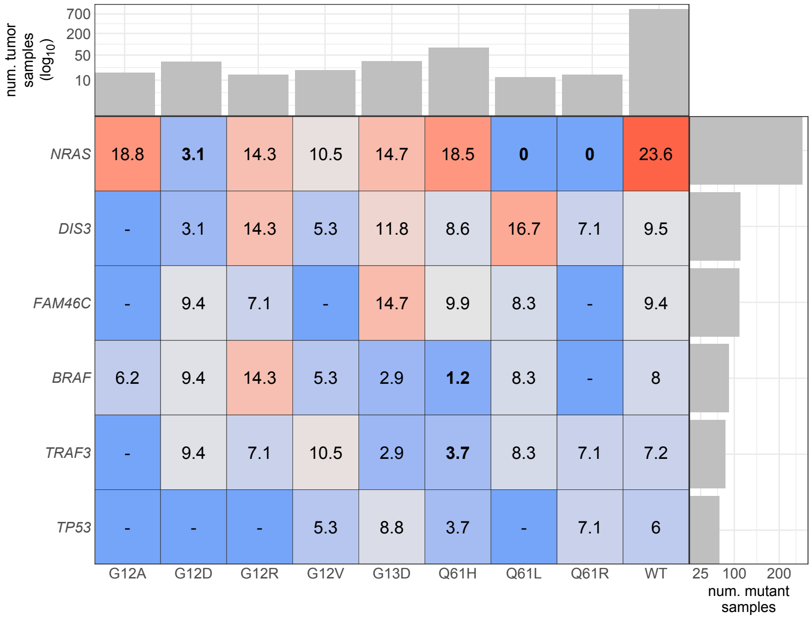

Supplementary Figure 6. The comutation frequencies between known MM driver genes or tumor suppressors and *KRAS* alleles. The color is correlated with the comutation frequency (the fraction of cells with the *KRAS* allele that also have a mutation in the other driver gene), indicated in each cell. Bold percent values indicate statistical significance of a comutation interaction (p-value < 0.01). The bar plot along the top indicates the number of samples with the *KRAS* allele, and the bar plot on the right indicates the number of samples with a mutation in the gene. n = 1,199 biologically independent MM tumor samples. Source data are provided in the Source Data file.

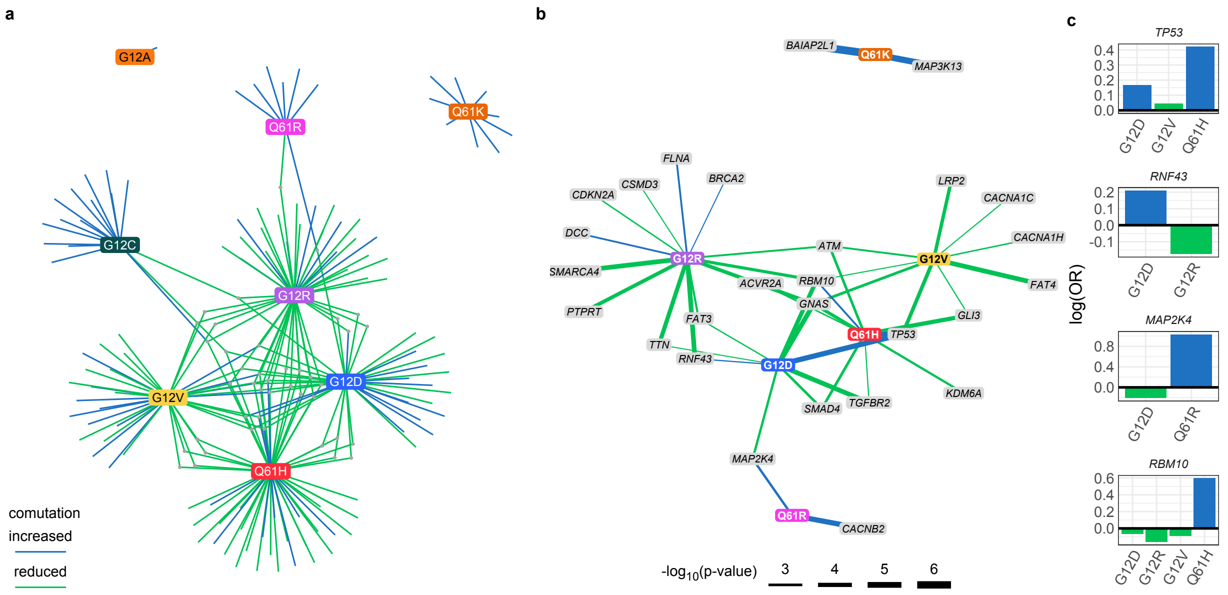

Supplementary Figure 7. The comutation networks of *KRAS* alleles in PAAD. a. The significant comutation network of the *KRAS* alleles in PAAD where each edge represents a comutation interaction between an allele and another gene (p-value < 0.01). The color of the edge indicates whether the interaction was an increase (blue) or decrease (green) in the frequency of comutation. Genes with multiple interactions are represented by a grey dot to disambiguate them from where edges intersect. **b.** A subset of the network shown in **a** of genes known to physically interact with *KRAS*, are in one of its canonical up- or downstream pathways, or are validated oncogenes or tumor suppressors. The width of the edge indicates the strength of the association. **c.** The log-odds of comutation between *KRAS* alleles and other genes that had detectable opposing comutation interactions with multiple alleles. n = 2,314 biologically independent PAAD tumor samples for the increased comutation analysis, and n = 1,395 biologically independent PAAD tumor samples for the reduced comutation analysis.

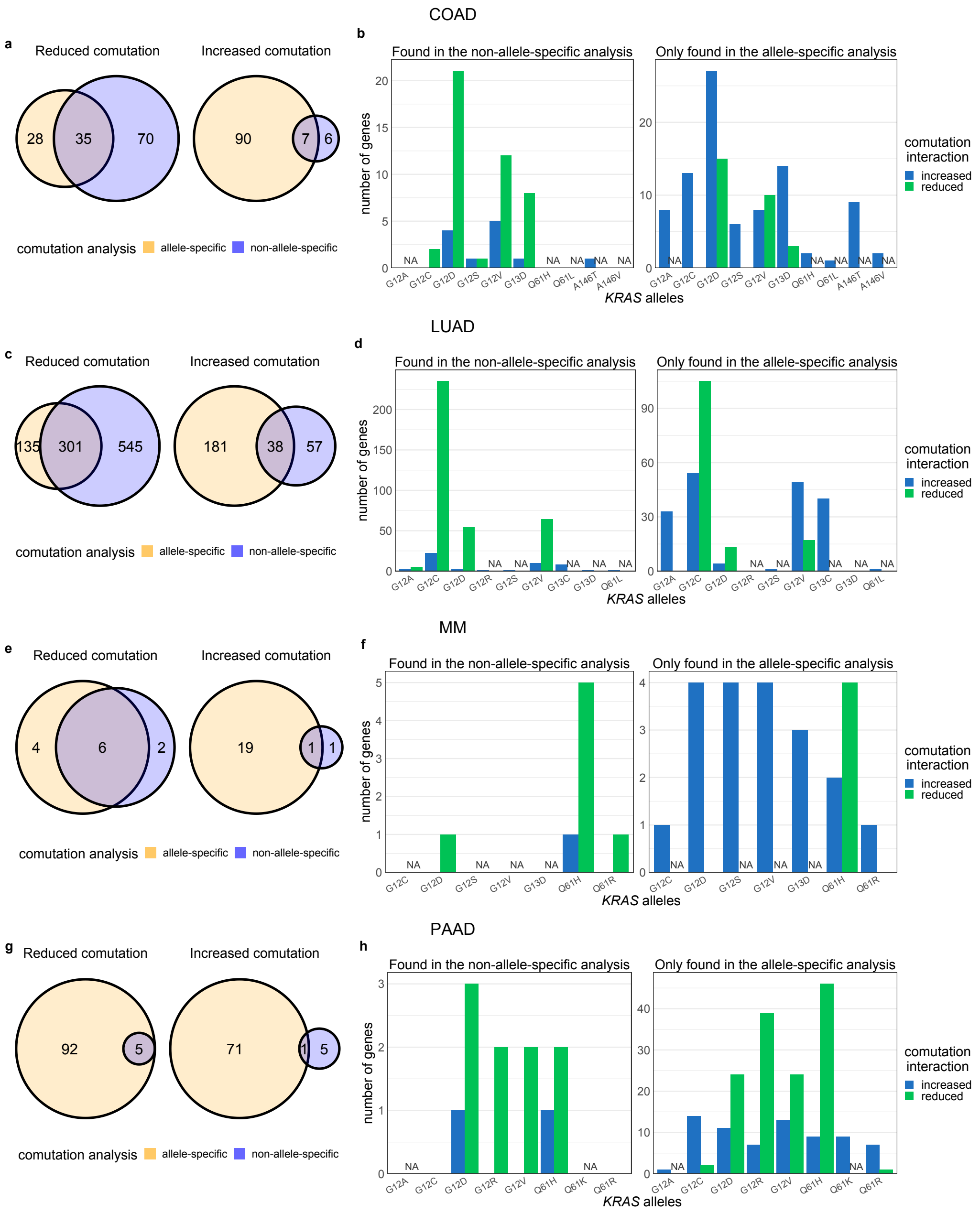

Supplementary Figure 8. Comparison of the number of genetic interactions between a *KRAS* allele-specific and non-allele-specific comutation analysis. The comutation analysis was conducted either treating all *KRAS* mutations as a single group ("non-allele-specific") or as different mutations ("allele-specific"). The Venn diagrams present the number of distinct and shared comutation interactions found in each analysis in (**a**) COAD, (**c**) LUAD, (**e**) MM, and (**g**) PAAD tumors. The Venn diagrams on the left represent reduced comutation interactions and those on the right represent increased comutation interactions. The bar-plots represent (left) the number of genetic interactions found for each *KRAS* allele in both the allele-specific analysis and the non-allele-specific analysis and (right) the number of genetic interactions found per *KRAS* allele that were not found in the non-allele-specific analysis in (**b**) COAD, (**d**) LUAD, (**f**) MM, and (**h**) PAAD tumors. Note that the non-allele-specific analysis tends to be biased towards identifying comutation interactions with the more common *KRAS* alleles, missing many interactions of the more rare alleles. Also, the non-allele-specific analysis of PAAD tumors identified very few interactions because only a small portion of samples are *KRAS* WT.

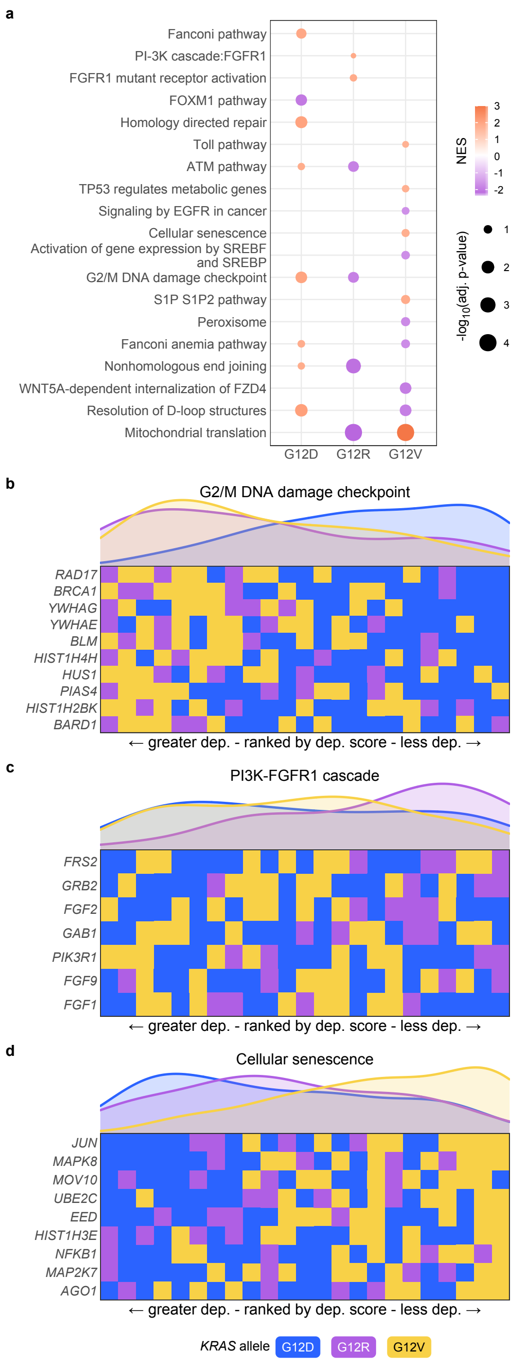

Supplementary Figure 9. Cellular processes enriched for greater or lesser genetic dependencies in PAAD cell lines separated by *KRAS* allele. a. Gene sets with significant enrichment for increased (lower dependency score; purple) or reduced (higher dependency score; orange) genetic dependency in PAAD cell lines. The size of the dot relates the FDR-adjusted p-value of the association, and the color indicates the strength of the enrichment. **b, c, d.** Heatmaps ranking the cell lines by dependency score of genes at the leading edge of enrichment for three gene sets in PAAD. Each row represents a gene and each cell represents a cell line colored by its *KRAS* allele. The cell lines were arranged in ranking order by their dependency score for the gene. Thus, each column indicates a rank. The line plots above the heatmaps indicate the representation (density) of each *KRAS* allele at each rank across the genes. n = 23 biologically independent PAAD cell lines. Source data are provided in the Source Data file.

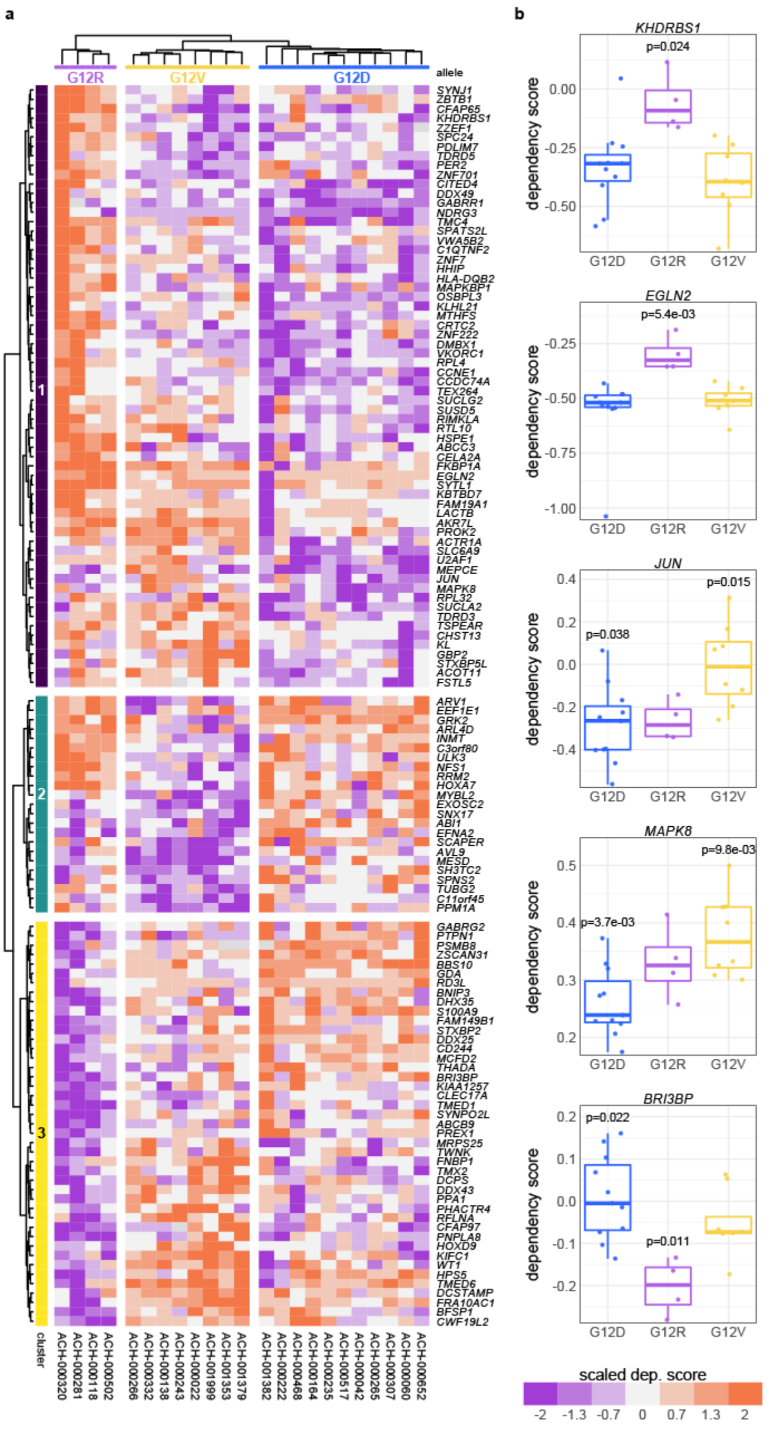

Supplementary Figure 10. Individual genes with differential genetic dependency by *KRAS* allele in PAAD cell lines. a. Clustered heatmaps of the genes that demonstrated differential genetic dependency amongst PAAD cell lines of different *KRAS* alleles. Each column is a cell line labeled by its DepMap identifier and each row is a gene. **b.** Examples of genes that demonstrated differential genetic dependency amongst cell lines of different *KRAS* alleles (*t*-tests; FDR-adjusted p-values). For the box plots, the box demarcations represent the 25th, 50th, and 75th percentiles, and the whiskers extend from the box to the largest and smallest data points at most 1.5 times the inter-quartile range away from the median. N = 23 biologically independent PAAD cell lines.
