# Supplementary material for: The origins and genetic interactions of KRAS mutations are allele- and tissue-specific
Source: Nat Commun. 2021 Mar 22;12:1808. doi: 10.1038/s41467-021-22125-z (PMC7985210; doi:10.1038/s41467-021-22125-z)
Supplement: Supplementary file 3 — Description of Additional Supplementary Files [file 41467_2021_22125_MOESM3_ESM.pdf]

Description of Additional Supplementary Files

File Name: Supplementary Data 1
Description: Data source summary.

File Name: Supplementary Data 2
Description: Targeted sequencing panel information.

File Name: Supplementary Data 3
Description: KRAS allele frequencies.

File Name: Supplementary Data 4
Description: Mutational signature compositions for each tumor sample.

File Name: Supplementary Data 5
Description: Mutational signature spectra.

File Name: Supplementary Data 6
Description: The predicted vs. observed KRAS allele frequencies for the most common alleles.

File Name: Supplementary Data 7
Description: The predicted vs. observed KRAS allele frequencies for all alleles detected in any cancer type.

File Name: Supplementary Data 8
Description: KRAS allele-specific comutation interactions.

File Name: Supplementary Data 9
Description: Non-allele-specific comutation interactions.

File Name: Supplementary Data 10
Description: Achilles data for the KRAS allele-specific genetic dependency interactions in COAD cell lines.

File Name: Supplementary Data 11
Description: Achilles data for the KRAS allele-specific genetic dependency interactions in PAAD cell lines.
