# Supplementary material for: The origins and genetic interactions of KRAS mutations are allele- and tissue-specific
Source: Nat Commun. 2021 Mar 22;12:1808. doi: 10.1038/s41467-021-22125-z (PMC7985210; doi:10.1038/s41467-021-22125-z)
Supplement: Supplementary file 5 — Reporting Summary [file 41467_2021_22125_MOESM5_ESM.pdf]

nature research

Corresponding author(s): Peter J. Park, Kevin M. Haigis

Last updated by author(s): Feb 12, 2021

Reporting Summary

Nature Research wishes to improve the reproducibility of the work that we publish. This form provides structure for consistency and transparency in reporting. For further information on Nature Research policies, see our Editorial Policies and the Editorial Policy Checklist.

Statistics

For all statistical analyses, confirm that the following items are present in the figure legend, table legend, main text, or Methods section.

n/a	Confirmed	
☐	☒	The exact sample size (*n*) for each experimental group/condition, given as a discrete number and unit of measurement
☐	☒	A statement on whether measurements were taken from distinct samples or whether the same sample was measured repeatedly
☐	☒	The statistical test(s) used AND whether they are one- or two-sided<br>*Only common tests should be described solely by name; describe more complex techniques in the Methods section.*
☐	☒	A description of all covariates tested
☐	☒	A description of any assumptions or corrections, such as tests of normality and adjustment for multiple comparisons
☐	☒	A full description of the statistical parameters including central tendency (e.g. means) or other basic estimates (e.g. regression coefficient) AND variation (e.g. standard deviation) or associated estimates of uncertainty (e.g. confidence intervals)
☐	☒	For null hypothesis testing, the test statistic (e.g. *F*, *t*, *r*) with confidence intervals, effect sizes, degrees of freedom and *P* value noted<br>*Give P values as exact values whenever suitable.*
☒	☐	For Bayesian analysis, information on the choice of priors and Markov chain Monte Carlo settings
☒	☐	For hierarchical and complex designs, identification of the appropriate level for tests and full reporting of outcomes
☐	☒	Estimates of effect sizes (e.g. Cohen's *d*, Pearson's *r*), indicating how they were calculated

Our web collection on statistics for biologists contains articles on many of the points above.

Software and code

Policy information about availability of computer code

Data collection	Data was collected using bash commands to access the web portals of the data sources used in this study.
Data analysis	The following software were used frequently throughout the analysis: bash (v4.2.46(2)-release), R (v4.0.1), Python (v3.7.4), Snakemake (5.4.5), SLURM (v20.11), conda (v4.7.5), 'renv' (v0.12.5), 'tidyverse' (v1.3.0), 'rlang' (v0.4.10), 'dplyr' (v1.0.4), 'magrittr' (v2.0.1), 'tibble' (v3.0.6), 'readr' (v1.4.0), 'ggplot2' (v3.3.3), 'tidyr' (v1.1.2), 'stringr' (v1.4.0), 'forcats' (v0.5.1), 'purrr' (v0.3.4), 'data.table' (v1.13.6), 'patchwork' (v1.0.0), 'cowplot' (v1.1.1), 'MASS' (v7.3-53), 'ProjectTemplate' (v0.9.2), 'memoise' (v1.1.0), 'mustashe' (v0.1.3), 'rmarkdown' (v2.6). The following software were critical to the mutational signature analysis: MATLAB (R2017b), SigProfiler (v1.5.0.0), 'boot' (v1.3-26), 'corrr' (v0.4.2), 'deconstructSigs' (v1.8.0). The following software were critical for the comutation analysis: Row-Column Exclusivity Test (implemented in 'wext' (v0.0.0.9002)), 'enrichr' (v2.1), 'tidygraph' (v1.2.0), 'ggraph' (v2.0.3), 'igraph' (v1.2.5), 'maftools' (v2.4.12), 'broom' (v0.7.4). The following software were critical to the analysis of genetic dependency: GSEA (v3.0), 'caret' (v6.0-86), 'pheatmap' (v1.0.12), 'dendextend' (v1.13.4).

For manuscripts utilizing custom algorithms or software that are central to the research but not yet described in published literature, software must be made available to editors and reviewers. We strongly encourage code deposition in a community repository (e.g. GitHub). See the Nature Research guidelines for submitting code & software for further information.

Data

Policy information about availability of data

All manuscripts must include a data availability statement. This statement should provide the following information, where applicable:

- Accession codes, unique identifiers, or web links for publicly available datasets
- A list of figures that have associated raw data
- A description of any restrictions on data availability

All data that support the findings of this study are publicly available from the cited sources. The compiled data is available upon request. The WGS, WES, and RNA

expression data of COAD, LUAD, and PAAD tumor samples are available on cBioPortal (http://www.cbioportal.org). The WGS, WES, and RNA expression data of MM tumor samples are available on the Multiple Myeloma Research Foundation's Research Gateway (https://research.themmrf.org). Additional WGS and WES of PAAD tumor samples generated by the ICGC were downloaded from ICGC data portal (https://dcc.icgc.org). The panel sequencing data of tumor samples are available through the dedicated GENIE instance of cBioPortal (https://www.cbioportal.org/genie/). All users must register and agree the AACR's terms of use before accessing the data. The Cancer Gene Census data was downloaded from the COSMIC website (https://cancer.sanger.ac.uk/census). The genetic dependency data (2020Q1) and cell line WGS and RNA expression data (generated by the CCLE) were downloaded from the DepMap web portal (https://depmap.org/portal/). Normal gene expression data was downloaded from the GTEx web portal (https://www.gtexportal.org). Normal protein expression data was downloaded from the Human Protein Atlas web portal (https://www.proteinatlas.org). The remaining data are available within the Article, Supplementary Information, or Source Data, or are available from the authors upon request.

Field-specific reporting

Please select the one below that is the best fit for your research. If you are not sure, read the appropriate sections before making your selection.

☒ Life sciences ☐ Behavioural & social sciences ☐ Ecological, evolutionary & environmental sciences

For a reference copy of the document with all sections, see nature.com/documents/nr-reporting-summary-flat.pdf

Life sciences study design

All studies must disclose on these points even when the disclosure is negative.

Sample size	This study only used previously available data and did not collect new data, thus we were not able to design the sample size of the study.
Data exclusions	Hypermutant COAD tumor samples were excluded from the comutation analysis because the high rate of passenger mutations would introduce noise into the calculations.<br>Cell lines with BRAF and other MAPK mutations were excluded from the analysis of allele-specific genetic dependency because were were unsure of whether to categorize them as KRAS WT or create another MAPK mutant group.
Replication	In this study, we only analyzed preexisting data and did not collect new data, thus we were not able to design replicate experiments.
Randomization	In this study, we only analyzed preexisting data and did not collect new data, thus we were not able to design the randomization protocols of the data generation processes.
Blinding	In this study, we only analyzed preexisting data and did not collect new data, thus we were not able to design the blinding protocols of the data generation processes.

Reporting for specific materials, systems and methods

We require information from authors about some types of materials, experimental systems and methods used in many studies. Here, indicate whether each material, system or method listed is relevant to your study. If you are not sure if a list item applies to your research, read the appropriate section before selecting a response.

Materials & experimental systems

n/a	Involved in the study	
☒	☐	Antibodies
☒	☐	Eukaryotic cell lines
☒	☐	Palaeontology and archaeology
☒	☐	Animals and other organisms
☒	☐	Human research participants
☒	☐	Clinical data
☒	☐	Dual use research of concern

Methods

n/a	Involved in the study	
☒	☐	ChIP-seq
☒	☐	Flow cytometry
☒	☐	MRI-based neuroimaging
